# Efficacy of cryotherapy plus topical *Juniperus excelsa* M. Bieb cream versus cryotherapy plus placebo in the treatment of Old World cutaneous leishmaniasis: A triple-blind randomized controlled clinical trial

**DOI:** 10.1371/journal.pntd.0005957

**Published:** 2017-10-05

**Authors:** Mohammad Mahdi Parvizi, Farhad Handjani, Mahmoodreza Moein, Gholamreza Hatam, Majid Nimrouzi, Jafar Hassanzadeh, Nasrin Hamidizadeh, Hamid Reza Khorrami, Mohammad Mehdi Zarshenas

**Affiliations:** 1 Research Center for Traditional Medicine and History of Medicine, Shiraz University of Medical Sciences, Shiraz, Iran; 2 Molecular Dermatology Research Center, Shiraz University of Medical Sciences, Shiraz, Iran; 3 Department of Traditional Persian Medicine, Shiraz University of Medical Sciences, Shiraz, Iran; 4 Department of dermatology, Shiraz University of Medical Sciences, Shiraz, Iran; 5 Medicinal Plants Processing Research Center, Shiraz University of Medical Sciences, Shiraz, Iran; 6 Department of Pharmacognosy, School of Pharmacy, Shiraz University of Medical Sciences, Shiraz, Iran; 7 Basic Sciences in Infectious Diseases Research Center, Shiraz University of Medical Sciences, Shiraz, Iran; 8 Research Center for Health Sciences, Institute of Health, Department of Epidemiology, Shiraz University of Medical Sciences, Shiraz, Iran; 9 Department of Parasitology and Mycology, Shiraz University of Medical Sciences, Shiraz, Iran; 10 Department of Phytopharmaceuticals (Traditional Pharmacy), School of Pharmacy, Shiraz University of Medical Sciences, Shiraz, Iran; Saudi Ministry of Health, SAUDI ARABIA

## Abstract

**Background:**

Cutaneous leishmaniasis is one of the highly prevalent endemic diseases in the Middle East and North Africa. Many treatment modalities have been recommended for this condition but success rates remain limited. Herbal remedies have also been used for treatment but evidence-based clinical trials with these products are sparse. In-vitro and in-vivo studies have shown the anti-leishmanial and curative effects of extract of fruits and leaves of *Juniperus excelsa* (*J*. *excelsa*). The aim of this study was to determine the efficacy of topical *J*. *excelsa* M. Bieb extract as an adjuvant to cryotherapy for the treatment of human CL.

**Materials and methods:**

This study was designed as a two-arm triple-blind randomized placebo-controlled clinical trial using a parallel design. Seventy-two patients with clinical diagnosis of CL confirmed by leishmania smears were allocated to receive either a topical formulation of leaf of *J*. *excelsa* extract (group A) or placebo (group B) for 3 months. Both groups received cryotherapy as baseline standard treatment. Patients were evaluated before and weekly after the intervention was initiated until complete cure.

**Results:**

Overall, 82% of patients in group A, experienced complete cure and 9% of them had partial cure. On the other hand, 34% in group B reported complete cure, while 14% of them had partial cure at the end of treatment protocol with a significant difference between the two groups (P< 0.001). The mean duration to healing of the lesions in patients who received *J*. *excelsa* extract was statistically significantly shorter than the placebo group (p = 0.04). No significant side effect was seen in the *J*. *excelsa* extract group except for mild to moderate local irritation after a few weeks in a few numbers of patients.

**Conclusion:**

The results of this study showed that topical *J*. *excelsa* extract can be used as an adjuvant treatment modality in addition to cryotherapy for accelerating the time to cure in addition to increasing the complete cure rate in CL.

**Trial registration:**

ClinicalTrials.gov IRCT2015082523753N1

## Author summary

Many people are afflicted in the world by cutaneous leishmaniasis (CL). The pathogen in this disease is an intracellular parasite. In 2007, The World Health Organization, defined leishmaniasis as a neglected disease. Many treatment modalities have been recommended for cutaneous leishmaniasis, but success rates remain limited. Experimental studies have shown the anti-leishmanial and curative effects of extract of fruits and leaves of *Juniperus excelsa* (*J*. *excelsa*), but there are no documentation in this regard in humans. This is the first randomized controlled clinical trial which evaluated the efficacy of the leaf extract of *J*. *excelsa* M. Bieb on human CL. According to the results of this present study, topical *J*. *excelsa* M. Bieb hydroalcoholic extract could be a good choice for treatment of CL in conjunction with cryotherapy. Using this extract along with cryotherapy can decrease the duration of CL treatment and also increase the success rate of CL treatment without any significant adverse effect.

## Introduction

Leishmaniasis refers to a set of diseases caused by intracellular protozoan of the genus *Leishmania* [1]. The pathogen in cutaneous leishmaniasis (CL) is an intracellular parasite. Rodents and canines are the common reservoir of the parasite. Unfortunately, humans are the causal host [1, 2].

Evidence suggests that CL is one of the oldest diseases throughout human history [3]. The concept of this disease goes back to 650 BC [4]. In medieval manuscripts, CL was described as early as the 10^th^ century by Avicenna as “Balkh sore” [5].

In 2007, the World Health Organization, designated leishmaniasis as a neglected disease, since more than 2 million people were afflicted in the world per year [6]. Ecological epidemiology of this disease is very diverse and dispersed in the world [7]. CL is endemic in 90 countries, mostly located in tropical, subtropical and southern Europe [1, 2]. Old World CL refers to this disease in the Middle East and North Africa [8]. Although, CL has been reported from 20 of 31 provinces in Iran, Fars province and Shiraz (the capital), in southwestern Iran, is one of main endemic areas for this disease in Iran [9, 10].

Many cases of cutaneous leishmaniasis are self-limiting and usually heal within a year [11, 12]. However, not all patients who have received treatment, eventually report the recovery and eradication of infection [13]. In the initial steps of treatment, it is very necessary to prevent chronicity of the lesions, which can cause malformed lesions on body surfaces [11, 12]. Reducing the severity of the infected wound and the patient's mental and emotional concerns [14, 15], and diminishing the reservoir and transmission of leishmaniasis are the most common reasons for treatment of this disease [16]. In some cases, CL ulcers can turn into large and disfiguring scars [17].

Several systemic and topical remedies are recommended for the treatment of CL. In the past few decades, antimonials, including meglumine antimoniate, have been the first line for the treatment of leishmaniasis. However, it is still accompanied by many complications[18]. Some studies have demonstrated that the efficacy of parenteral antimonials for the treatment of CL in Iran is 60–80 percent [19]. Intralesional injection of meglumine antimoniate, is also used for the treatment of CL and the cure rate reports of this route in Iran is 55–75% [20, 21]. However, since systemic antimonials have many side effects, clinicians face poor patient compliance [22–24].

Cryotherapy is a common treatment for CL, especially when the lesions are: non-complicated, less lymphocutaneous, less than three months duration, small lesions less than four in number, and for those who cannot receive systemic treatment [21, 25, 26]. Generally, cryotherapy with liquid nitrogen includes a cycle of freeze-thaw-freeze resulting in intracellular ice to form, which destroys the cell leading to localized ischemic necrosis [27]. This procedure decreases the local tissue temperature and metabolism which may result in cryonecrosis and can destroy the amastigotes and activate an immune response produced by the liberation of antigenic substances [28, 29].

Several studies have demonstrated that combination of cryotherapy with meglumine antimoniate increases the cure rate of leishmaniasis [21]. Besides, the side effects and limitation for using antimonials for treatment of CL, patient compliance has also led to its limited use [30]. However, use of adjuvant drugs could be useful for improving wound healing and increasing the rate of healing [31].

*Juniperus excelsa* (Cupressaceae) is divided into two main subspecies including *excelsa* M. Bieb and *polycarpus*, *the first* is called Persian Juniper [32, 33]. *Juniperus excelsa* M. Bieb (*J*. *excelsa)* is one of the most common genera in the related family [34]. It is called “*Urs”* or “*Abhol”* in famous textbooks of traditional Persian medicine, such as Avicenna's (980–1037 AD) [35], Canon of Medicine or the “Storehouse of Medicaments” by Aghili Alavī Shirazi (1670–1747 AD) [36]. Rhazes (865–925 AD) [37], who is known as a pioneer in the field of dermatology [38], mentioned its different medicinal uses, including its application as a remedy for treatment of old wounds and infected wounds [39]. Fruits, leaves, wood and extracts of parts of these plants have been used for medical, sanitarian and cosmetic purposes [40, 41]. *J*. *excelsa* essential oil has several compounds with proved anti-inflammatory, anti-toxicity and wound healing effects, which have been reported in several studies [42, 43]. In addition, there is some evidence that shows that this plant has anti-leishmaniasis activities in *in-vitro* and *in-vivo* studies [44]. Therefore, the aim of this study was to assess the efficacy of *J*. *excelsa* 5% hydroalcoholic extract cream in conjunction with cryotherapy versus cryotherapy plus placebo in search of an adjuvant treatment for CL.

## Materials and methods

### Design of the study and ethics statements

This study was designed as a triple blinded randomized controlled clinical trial. The Ethics Committee of Shiraz University of Medical Sciences approved the protocol (IR.SUMS.REC.1394.91) on August 23, 2015. This clinical trial was then registered at Iranian Registry of Clinical Trials (IRCT) website by IRCT2015082523753N1 code (http://en.search.irct.ir/view/25321).

All patients were adult (above 18 years old) and were aware of the trial plan. Written informed consent was obtained from all participants prior to enrollment and patients were free to withdraw from the project at any time. All patient data were also anonymized.

### Sample size and study population

This study was performed at the Molecular Dermatology Research Center and *Shohadaye-Enghelab* Health Center, Shiraz University of Medical Sciences, Shiraz, Iran from September 2015 to September 2016.

Patients 18 to 70 years old with a maximum number of four lesions, ulcer size with maximum diameter of five centimetres, duration of lesions no more than four months, and patients receiving no anti-leishmania treatment remedies during the past four months were eligible for inclusion. Pregnant and lactating women, patients with regional adenopathy and those who had face involvement were excluded from the trial.

It was calculated that a minimum sample size of 25 patients per group was required to demonstrate a difference of 35% (80% versus 46%) in complete cure rate between the groups by accepting a two-tailed alpha error of 0.05 and a beta error of 0.20. To allow for incomplete follow up, with estimated lost-to-follow-up, it was decided to randomize 72 patients.

A total of 72 patients with positive skin smear for CL were enrolled. Skin scraping was taken from the margin of the active lesion, smeared on a glass slide, fixed with methanol and then, Giemsa stained and microscopically inspected for amastigotes of leishmania.

### Randomization and blinding

Random allocation software Ink (Version 1.0, May 2004) was used to create a randomization table by a block size of four. Therefore, the patients were allocated to A and B groups according to the randomization table, respectively. This study was a triple blind trial. The herbal extract and placebo were delivered to the patient in a similar laminate tube marked as “A” or “B”. A dermatologist assessed the lesions of both groups, blindly. Also, the person who performed the statistical analysis was blinded and was only aware of the groups by the allocations “A” or “B”.

### Data collection

Demographic data including sex and age of the participant, location and number of the ulcers, size of the lesions, and duration of lesions were recorded for all patients. Visits of the patients were done weekly and treatment outcomes and changes in size of ulcers were recorded.

### Interventions and follow up

All patients received cryotherapy as a standard and baseline treatment for CL. Cryotherapy was done by liquid nitrogen (−195°C), once weekly for each lesion with a margin of about 1–2 millimetres with a cotton swap. The freezing time was up to 20 seconds in each visit. Based on the improvement or worsening of lesions, the assessor dermatologist decided if the patient needed further cryotherapy sessions or not.

At the time of the first session of cryotherapy, patients in group A received 5% hydroalcoholic extract of leaves of *J*. *excelsa* as a topical cream, in addition to cryotherapy, three times daily. Patients in group B received placebo cream three times daily (although a small number of patients reported twice daily applications), in addition to cryotherapy. Following applying the cream each time, the patients were recommended to wash the site of the lesion with water and soap to prevent possible secondary infection. None of the patients were prescribed either topical or oral antibiotics at the first week of intervention. Follow-ups for each patient were continued for three months (12 weeks). For compliance issues, each patient was asked to come back weekly with the container that was given to him/her on the previous week and the investigator could assess the approximate amount of topical treatment that was used.

At any time, after eight-weeks of starting therapy, if there was evidence of deterioration of the lesion, the patient was treated with other available remedies due to ethical reasons. In this condition, the case was considered as failure to treatment. Although, a long-term follow up of the patients was not included in the initial plan of our study, we decided to follow up these patients either by personal visits or by telephone for up to six months after completing the study to check for possible recurrence of the lesions.

### Drug and placebo preparation

Leaf of *J*. *excelsa* was used to prepare the cream. Leaves of the plant was collected from *Geno* Biosphere Reserve, Bandar Abbas, Hormozgan province, south of Iran. Authentication of the plant was performed by a botanist at School of Pharmacy, Shiraz University of Medical Sciences with a specified voucher number (NO: PM 852).

The hydroalcoholic extract of the leaf was yielded using percolator apparatus. The obtained extract was then concentrated and dried. Both *J*. *excelsa* extract (JE) and placebo were made in cream (oil in water) form.

The amount and type of ingredients in both the JE and placebo creams were the same and included beeswax, thickeners agent, petrolatum, paraffin and ethanol (as solvent for dissolving the dry extract ≈ 5% of aqueous phase in cream), except for 5% of the extract which was added to the main cream. To create a similarity in the appearance of the placebo and JE cream, a standard green color powder was used in the placebo cream to make it similar to the JE cream. All steps in the preparation of the cream were performed under sterile air inside a laminar airflow cabinet. All equipments associated with the production of creams, including glass supplies, laboratory equipment, digital scales, Ben Murray, and homogenizer were previously cleaned by ethanol 70%. The JE and placebo creams were distributed to patients in similar 50g white laminated tubes.

### Phytochemical assessment

#### Volatile oil extraction

In this study, both employed extract of *J*. *excelsa* and finished product (J. excelsa 5% cream) were analyzed in regard of the volatile constituents. To this, the extract and prepared cream were individually subjected to hydrodistillation for 3 hours using a Clevenger-type apparatus. Respective essential oil samples were dried and kept in 4°C for further steps.

#### Analysis of the essential oil

Initially GC/FID oil analysis was carried out to find a proper analytical condition. This process was performed on a gas chromatograph Agilent technologies model 7890A (USA) apparatus attached to HP-5 column (25 m × 0.32 mm, 0.52μm film thickness) and connected to a Agilent technologies (USA) flame ionization detector (FID). Nitrogen gas was employed as carrier gas with a flow rate of 1 ml/min (split ratio was 1:30). The injector temperature was 250°C, and detector temperature was 280°C, while column temperature was linearly programmed from 60 to 250°C (at rate of 5°C/min) and held for 10 min at 250°C. Solutions of anhydrous and diluted essential oil samples from cream and extract were consecutively injected. The above method was considered for GC/MS analysis. The process was carried out via using Agilent technologies model 7890A gas chromatograph (USA) connected to a mass detector (Agilent technologies model 5975C- USA). The GC was equipped with a HP-5MS capillary column (phenyl methyl siloxane, 30 m × 0.25 mm i.d., Agilent technologies). Helium was employed as carrier gas with the same flow rate as for GC/FID. The mass spectrometer was acquired in EI mode (70 eV) in a mass range of 30–600 m/z. The interface temperature was 280°C. Identification and analysis of components was based on the comparison of their mass spectra with Willey (nl7) and Adams libraries spectra as well as with those cited in literature.

#### Determination of total phenol and flavonoid content (nonvolatile constituents) in prepared cream

Total flavonoid content in *J*. *excelsa* cream was determined using an Aluminium chloride colorimetric assay (A modified Dowd method). A solution of 5 ml Aluminium trichloride (2%) (Sigma Aldrich- USA) in methanol (Merck- Germany) was mixed with same volume of different concentrations of quercetin (as control). Absorption was read at 415 nm (PG instrument T90 spectrophotometer- Germany) after 10 minutes against a blank sample (5 ml extract solutions with 5 ml methanol in the absence of AlCl_3_). Using a preparing quercetin (Sigma-Aldrich) standard curve (concentrations ≈ 0–80 mg/L), total flavonoid content of samples were determined. The mean of three readings for each sample was considered and expressed as mg of quercetin equivalents (QE)/g of dry plant leaves [45].

Total phenol content in finished product was carried out using Folin–Ciocalteu method. To this, 0.5 ml aliquots of 0.024, 0.075, 0.105 and 0.3 mg/ml methanol Gallic acid (Merck- Germany) solutions were mixed with 2.5 ml Folin Ciocalteu reagent (Sigma Aldrich- USA) and 2 ml (75 g/l) sodium carbonate. Absorption (765 nm) was read after 30 min at 20°C, and calibration curve was drawn. Approximately, 0.5 ml cream (10 g/L) was mixed with the above reagents. Absorption was read (after 1 hour) for the determination of JE cream phenolics. All determinations were done in triplicate. Total content of phenolic compounds in the methanol extracts in Gallic acid equivalents (GAE) was calculated via the following formula:

C=c.V/m

Where: “C” is the total content of phenolic compounds, mg/g extract, in GAE; “c” is Gallic acid concentration established from the calibration curve, mg/ml; “v” is the extract volume, ml; and “m” is the methanol extract weight, mg [46].

### Evaluation of the lesions

Evaluation of the lesions was carried out weekly (the interval between each visit was 7± 2 days). The improving rate was determined by measuring the lesion size at baseline and also weekly by a scaled ruler (in millimeter) up to the time of cure or the end of the study. The size of the lesions were measured in two perpendicular directions by the dermatologist. The area of the lesion was calculated with ImageJ^®^ Software 1.44p (An open platform for scientific image analysis, Wayne Rashband, National Institute of Health, Bethesda, Maryland, USA). Clinical response to treatment was defined based on the following criteria:

1. Complete cure (decrease in lesion size with re-epithelization > 90%)
2. Partial cure (decrease in lesion size with re-epithelization between 50–90%)
3. No improvement (decrease in lesion size and re-epithelization < 50%).

### Preparation of the smear and diagnosis of leishmaniasis

Cytological smears were prepared by scraping of the skin lesions with a scalpel. Multiple smears were made on slides and were both air dried and alcohol fixed and then stained by the Wright method.[47, 48] Review of cytological smears was conducted by a single expert laboratory personnel. Microscopic examination showed the amastigote forms of *Leishmania* in magnification, ×200.

### DNA extraction and nested PCR

Leishmania species identification was determined using nested PCR method amplifying the kinetoplastid DNA from Giemsa-stained smears of CL lesions. DNA was extracted using phenol–chloroform–isoamyl alcohol as previously described [49]. AccuPrep^®^ Genomic DNA Extraction Kit (Bioneer, Daejeon, Korea), was performed to extract genomic DNA from each clinical sample, according to the manufacturer’s instructions. Special primers related to variable regions of kDNA were used in PCR analysis as previously described [49, 50].

External primers CSB2XF and CSB1XR in the first round of PCR and internal primers 13Z and LiR in the second round of PCR were applied. The PCR products were analyzed by 1.5% agarose gel electrophoresis. We used PCR products on the promastigote cultures of the reference strains of *L*. *infantum*, *L*. *major* and *L*. *tropica* as positive controls. Nested PCR analysis resulted in a fragment of 680 bp [49, 51].

### Statistical analysis

Statistical analysis was conducted using the SPSS version 22 (IBM Corporation, Armonk, NY). In addition, R Statistical Software (version 3.3.1; R Foundation for Statistical Computing, Vienna, Austria) was carried out to drawing a plot for comparing the changing size of the lesions in both groups over time. Normally distributed quantitative variables were demonstrated as mean ± standard deviation (SD). The normality distribution of the quantitative variables was investigated using the Kolmogorov-Smirnov test. Chi-square was used for assessing the relationship between categorical variables and groups. Generalized Estimating Equation model (GEE) was used for modeling the response (size of the lesion) with time and to assess the effect of the JE on it. Finally, multinomial logistic regression was used for estimating the odd ratio of JE effectiveness. The p-value of less than 0.05 was considered as significant.

## Results

### Patient enrolment

**Fig 1:**
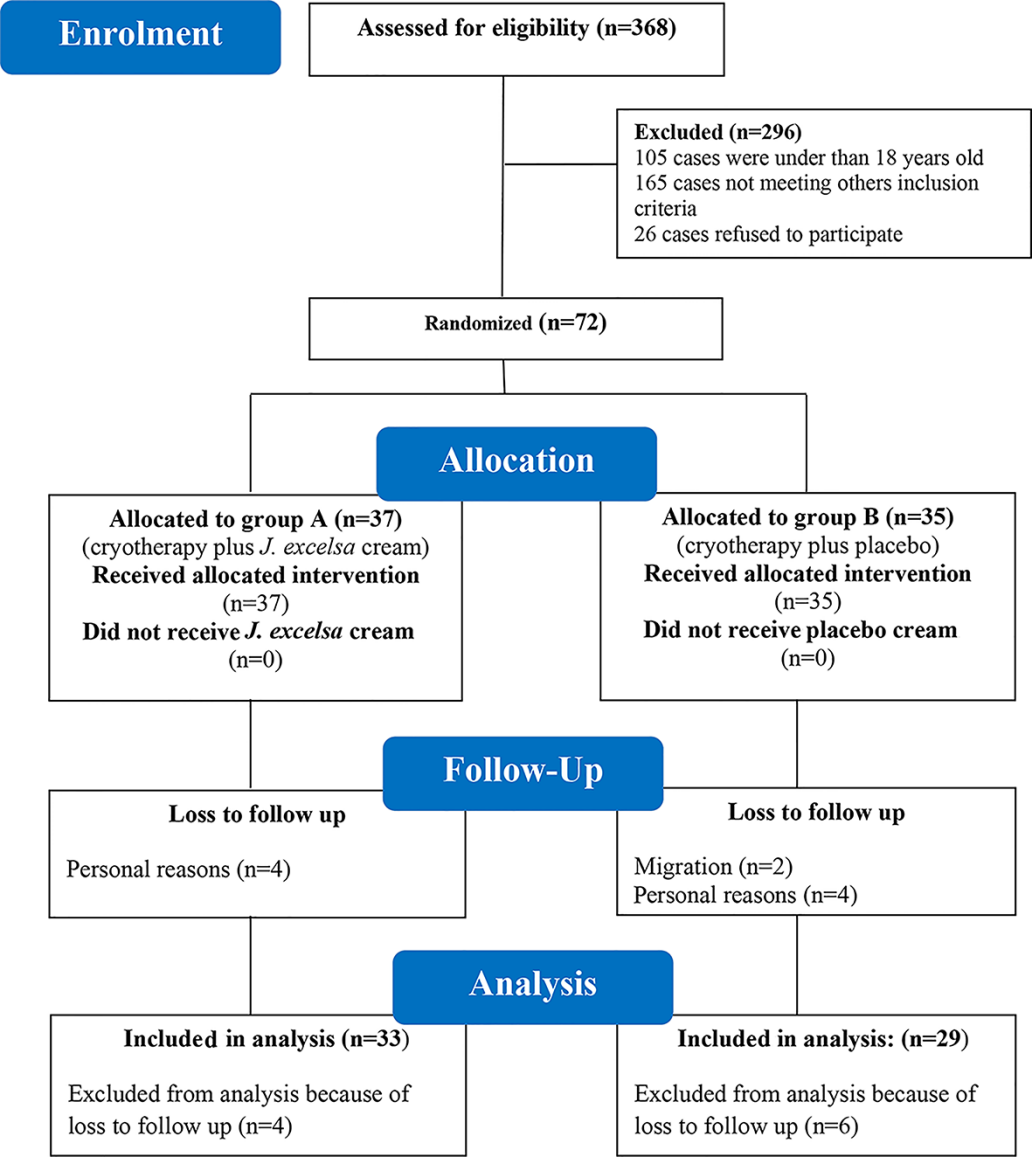
CONSORT chart of the clinical trial of therapeutic effect of *Juniperus excelsa* M. Bieb extract cream on cutaneous leishmaniasis.

From a total number of 368 visited patients, 72 were enrolled in the study, based on our inclusion criteria. Overall, 10 out of 72 patients who were enrolled in the study, left the study. Therefore, 62 patients including 33 in group A (21 males and 12 females) and 29 in group B (16 males and 13 female) completed the study protocol and were analyzed at the end of the study. Because a few patients used the topical cream two times daily for a few days due to forgetfulness or busy job schedule, we used intention to treat method for analyses of the data. Detailed information about the study CONSORT flow chart is shown in Fig 1. All remaining patients were in-line with the protocol of the study.

### Demographic characteristics

The gender distribution showed a male majority in both groups, however, the gender difference between the two groups was not statistically significant.

The mean ± SD of age was 38.91±13.49 years in group A and 42.10±14.54 years in group B, presenting no statistically significant differences between both groups. There were no statistically significant difference for the other baseline demographic data between the two groups as shown in Table 1.

**Table 1:** Demographic characteristics of CL patients in both groups (Group A and Group B).

Variables	Cryotherapy plus JE	Cryotherapy plus placebo	P-value
	(Group A)	(Group B)	
**Sex**			0.49
Male	21 (67%)	16 (55%)	
Female	12 (36%)	13 (45%)	
**Age** (year, mean± SD)	38.91±13.49	42.10±14.54	0.437
**Marriage status**			0.24
Single	9 (27%)	12 (41%)	
Married	24 (73%)	17 (57%)	
**Educational status**			0.53
Under-Diploma	21 (64%)	17 (59%)	
Diploma	5 (15%)	7 (24%)	
Associate Degree	3 (9%)	2 (7%)	
Bachelor Degree	4 (12%)	3 (10%)	
**Location of the lesions**			0.61
Upper extremity	19 (58%)	16 (55%)	
Lower extremity	11 (33%)	8 (28%)	
Both upper and lower extremities	3 (9%)	5 (17)	
**Number of the lesions**			0.65
1	18 (55%)	17 (57%)	
2	8 (24%)	5 (17%)	
3	5 (15%)	3 (10%)	
4	2 (6%)	4 (14%)	
**PCR characterization of microscopic positive samples**			0.85
*Leishmania major*	28 (85%)	24 (83%)	
*Leshmania infantum*	0 (0%)	1 (3%)	
*Leshmania tropica*	0 (0%)	0 (0%)	
Negative result of PCR	5 (15%)	4 (14%)	
**Duration between time of lesion occurrence and the time of first visit for treatment** (month, mean± SD)	1.56±0.74	1.44±0.83	0.34
**Vertical diameter of lesion on first visit**(mm, mean± SD)	21.38± 7.87	20.41±7.95	0.65
**Horizontal diameter of lesion on first visit** (mm, mean± SD)	16.66± 5.63	17.52± 7.05	0.6
**Area of the lesion** (mm^2^, mean± SD)	306.26±226.60	336.95±303.32	0.94

**JE:** *Juniperus excelsa* M. Bieb *extract*, **mm:** millimeter; **mm**^**2**^: square millimeter; SD: standard deviation; PCR: Polymerase chain reaction

The majority of patients had only one lesion in both groups (18 in group A vs. 17 in group B). Also, most patients had the lesion in the upper extremity (19 in group A vs. 16 in group B).

Number of lesions, duration of lesions, baseline vertical diameter size, baseline horizontal diameter size and the baseline area of lesions showed no statistically significant differences between both groups (Table 1).

### Leishmania species identification

**Fig 2:**
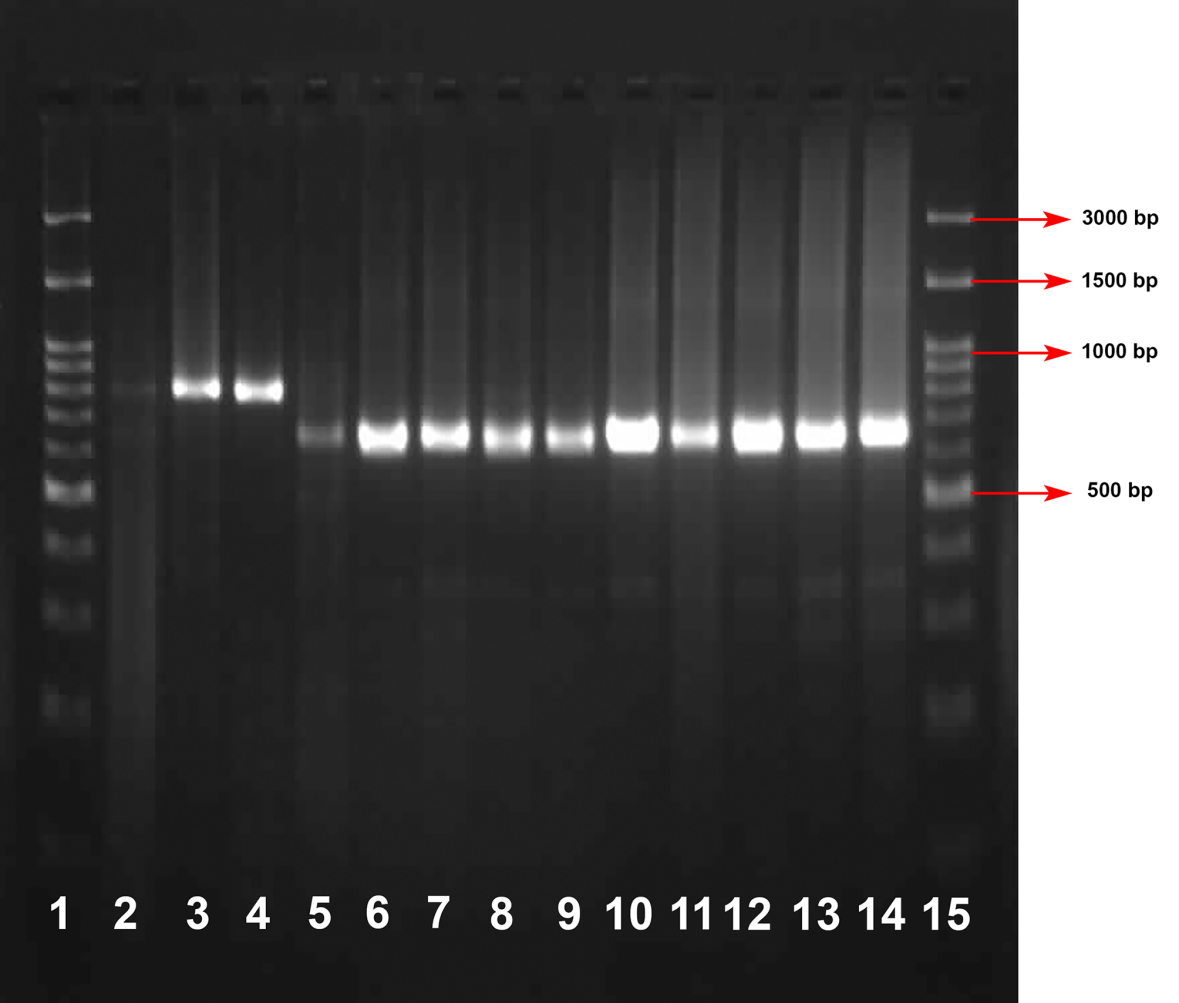
Electrophoresis of PCR products of DNA extracted from positive smears. The 15 lanes are shown in this figure and consist of: ladder lanes (1 and 15); weakly positive (lane 2); positive control of *L*. *infantum* (lane 3); positive control of *L*. *major* (lane 14); Patients samples (lanes 4–13).

An example of a nested PCR analysis is shown in Fig 2. All patients in both groups were infected with *L*. *major* except one in group B, who was infected by *L*. *infantum*. Also five and four patients in group A and B, respectively, had negative results in PCR for leishmaniasis.

### Response to the treatment

Overall, 27 out of 33 (82%) patients in group A, experienced a complete cure and three of them (9%) had partial cure. On the other hand, 10 out of 29 patients (34%) in group B reported complete cure, while four of them (14%) had partial cure. Three patients in group A and 15 patients in group B were designated as treatment failures. The details are shown in Table 2.

**Table 2:** Outcome of the treatment in both groups (Group A and Group B).

Variable	Cryotherapy plus JE	Cryotherapy plus placebo	P-value
	(Group A)	(Group B)	
**Result of treatment** N (%)			<0.001*
Complete cure	27 (82%)	10 (34%)	
Partial cure	3 (9%)	4 (14%)	
Failure to treatment	3 (9%)	15 (52%)	
**Duration to cure**	6.48±2.96	8.72±3.34	0.04*
(mean± SD of weeks)			
**Drug reaction** N (%)			0.055
No	28 (85%)	29 (100%)	
Yes	5 (15%)	0 (0%)	
**Number of cryotherapy sessions in patients with complete cure** (mean± SD)	3.85±2.03	6.54±3.35	0.026*

*Significant at 5%, **JE:** *Juniperus excelsa* M. Bieb *extract*, **SD:** standard deviation

The mean duration between the starting point of treatment to the time of complete cure in group A was significantly shorter than group B. Also, the average number of cryotherapy sessions in patients with complete cure in group A was 3.85±2.03 as compared to 6.54±3.35 sessions in group B, as shown in Table 2.

**Fig 3:**
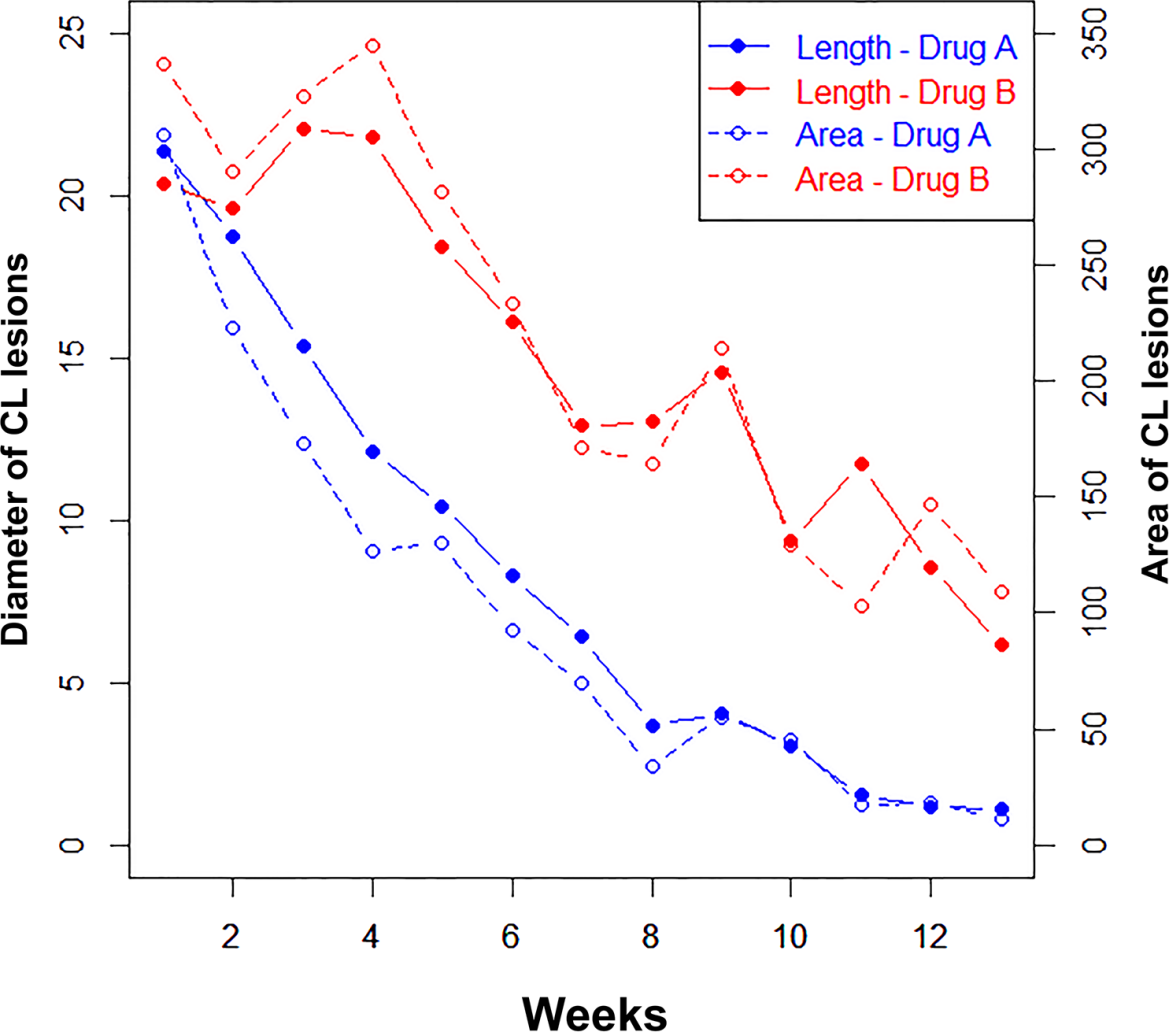
Changes in the size of CL lesions in both groups during three months. The numbers on the left side of the chart indicate the changes of length of the ulcers (mm) and the numbers on right side of the chart indicate the area changes (mm^2^) in the duration of treatment (12 weeks).

**Fig 4:**
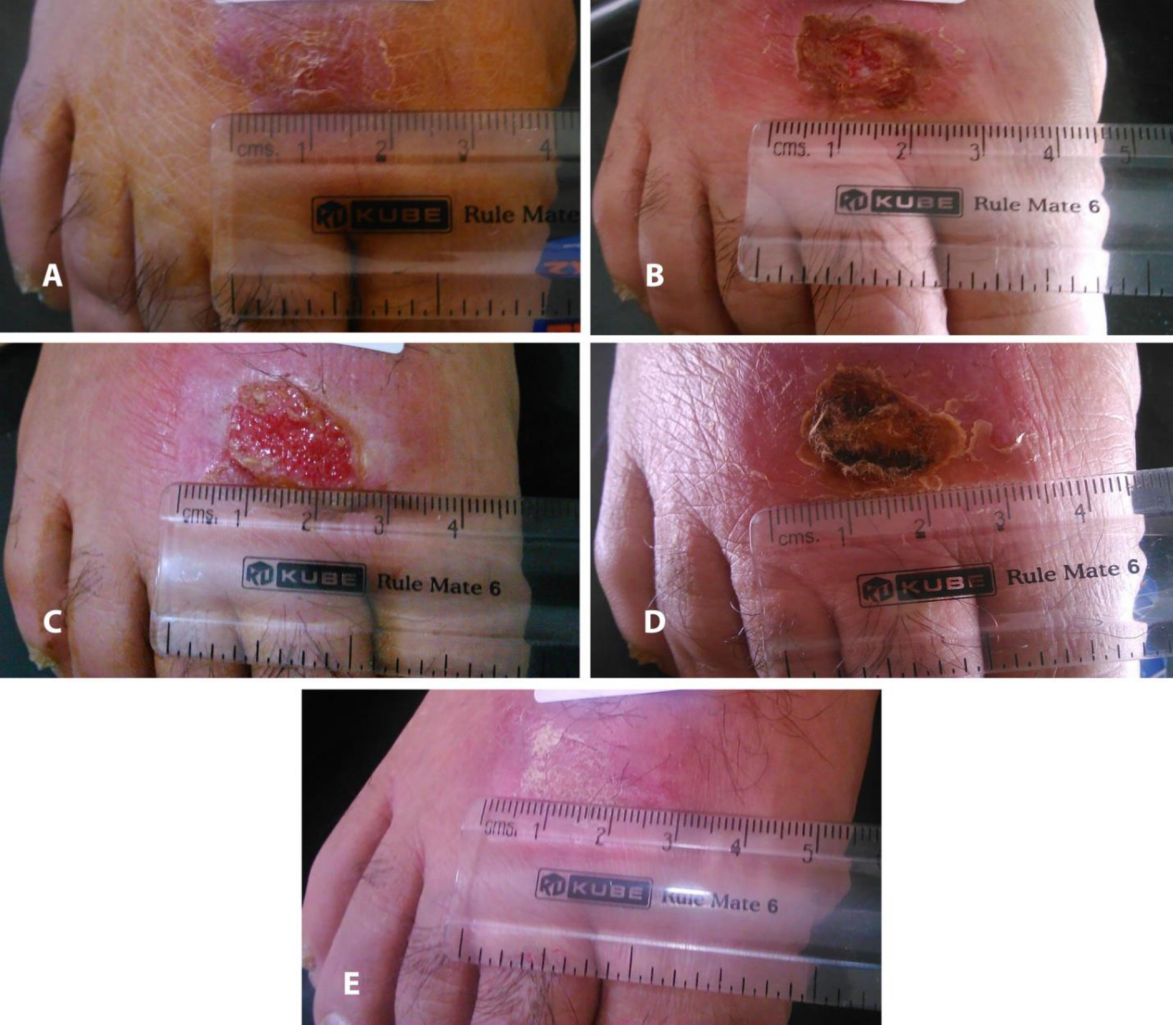
CL patient who was cured in group A. (A)Before treatment, (B)after one week, (C) after two weeks, (D)after three weeks, (E) after five weeks.

The overall area of the lesions in group A and B were 100.89±14.58 mm^2^ and 217.62±31.73 mm^2^, respectively. The GEE analysis revealed that the area of lesions of CL decreased during the 12 week treatment plan in patients in both JE and placebo groups, showing that the effect of time was statistically significant (coefficient parameter estimation: 21.36±3.17; p<0.001). Moreover, our findings indicated that the time interval to healing in the JE group was shorter than in the placebo group and this difference was statistically significant (coefficient parameter estimation: 116.96±35.35; p<0.001). Detailed information about estimation of this parameter by GEE is shown Fig 3. In addition, multinomial logistic regression revealed that the rate of complete cure in patients who received the JE was 13.5 times in comparison with those who received placebo (OR = 13.50, 95% CI 3.210–56.770, P-value< 0.001). Fig 4 shows complete cure for the patient who received JE.

In long term follow-up using personal visits and telephone contacts, all patients had complete cure after six months and none of them showed or reported any evidence of disease relapse.

### Safety profile

Five out of 33 patients in group A, experienced local irritation including redness and itching after about 5 weeks of JE application (delayed local reaction to the herbal extract), but none of the reactions were severe. Only one patient who returned on the sixth week of treatment with moderate erythema, redness and itching in her arm stopped using the medication although she had achieved complete cure at that time. One of the patients with irritation due to the JE failed treatment. The other three patients continued using the JE cream as they experienced mild degrees of redness and itching.

### Phytochemical content

Considering that the JE has been evaluated for anti-leishmanial activity in a human study, it was introduced into the semi-solid cream product. Therefore, in order to achieve some therapeutic responses in terms of repeatability, some of the chemical compounds and metabolites in final product containing 5% of the JE was evaluated. These metabolites were volatile compounds and phenolic compounds as well as total flavonoids in the JE cream product.

GC/MS analysis of the plant extract volatile constituents revealed a total of 28 constituents (Table 3). Major compounds were sesquiterpenes (≈ 75% of total identification). On the other hand, most fractions of metabolites in volatile profile extracted from prepared cream were hydrocarbons (38.63%), phenols (34.74%) and sesquiterpenes (21.29%) (Table 3).

**Table 3:** Volatile constituents of both the plant methanol extract and prepared JE cream^1^.

No.	Component	Area % (Extract)	Area % (Cream)	KI^C^	KI^R^
1	α-Pinene	-	0.24	935	939
2	Limonene	-	0.21	1030	1029
3	Linalool	1.02	0.34	1100	1096
4	trans-Pinocarveol	-	0.27	1142	1139
5	Borneol	0.36	0.38	1168	1168
6	α- Terpineol	0.31	-	1193	1188
7	n-Dodecane	-	0.45	1200	1200
8	n-Decanol	-	0.19	1272	1269
9	Thymol	0.71	-	1291	1290
10	n-Tridecane	-	0.53	1300	1300
11	Carvacrol	1.55	-	1301	1290
12	α-Cubebene	0.5	-	1353	1348
13	Hexyl hexanoate	-	0.3	1387	1383
14	n-Tetradecane	-	0.83	1400	1400
15	β-Funebrene	-	1.37	1419	1414
16	trans-Caryophyllene	-	0.32	1425	1419
17	α-Humulene	-	0.37	1459	1454
18	n-Dodecanol		2.74	1477	1472
19	γ-Muurolene	0.53	0.22	1478	1479
20	α-Amorphene	0.71	-	1481	1484
21	Germacrene D	0.53	-	1497	1485
22	n-pentadecane	-	1.63	1503	1500
23	α-Muurolene	1.72		1505	1500
24	ɣ-Cadinene	1.51		1519	1513
25	Butylated hydroxytoluene	**-**	**34.74**	1524	1515
26	Methyl dodecanoate	1.45	-	1525	1525
27	Δ-Cadinene	4.42	0.65	1528	1523
28	Elemol	**13.36**	0.7	1555	1549
29	Germacrene B	-	0.45	1564	1561
30	Dodecanoic acid	1.78	-	1570	1566
31	Spathulenol	0.6	0.73	1582	1578
32	Caryophyllene oxide	0.83	-	1588	1583
33	Ethyl dodecanoate	0.42	-	1593	1595
34	n-Hexadecane	-	1.37	1600	1600
35	β-Oplopenone	1.23		1613	1607
36	Cedrol	**-**	**13.66**	1613	1619
37	α -Cadinol	**7.9**	1.17	1636	1640
38	β -Eudesmol	**7.51**	0.79	1647	1650
39	α-Eudesmol	**30.02**	0.46	1660	1653
40	n-Heptadecane	1.16	0.87	1698	1700
41	Methyl tetradecanoate	0.91		1723	1723
42	n-Octadecane	-	0.64	1797	1800
43	n-Hexadecanol	**-**	**19.99**	1886	1875
44	n-Nonadecan	-	0.24	1898	1900
45	Methyl hexadecanoate	0.84	-	1923	1921
46	Hexadecanoic acid	1.26	-	1958	1960
47	n-Eicosane	0.69	0.19	1996	2000
48	n-octadecanol	**-**	**8.72**	2085	2077
49	Methyl linoleate	0.39	-	2091	2085
	**Total identification**	**84.22**	**95.76**	-	-
	**Monoterpenes**	**3.95**	**1.1**	-	-
	**Sesquiterpenes**	**75.02**	**21.29**	-	-
	**Phenols**	**-**	**34.74**		
	**Hydrocarbons**	**5.25**	**38.63**	-	-

^1^The significance of bold is to present the most abundant constituents. Compounds have been identified by combination of both mass spectra and retention indices. RI represents the retention indices which were calculated against C8-C24 n-alkanes in the mentioned column. Compounds have been sorted with respect to retention indices on HP-5 MS capillary column.

Total phenolic compounds and total flavonoids in the prepared JE cream were 1.85±0.014 mg Gallic acid equivalent/g and 0.31±0.006 mg quercetin equivalent/g, respectively. Accordingly, the amount of phenolic compounds and total flavonoids in the tube containing 50 g of prepared JE cream were 93.03±1.13 mg and 16.31±0.05 mg, respectively.

## Discussion

Few studies have shown the in-vitro leishmania promastigocidal effect of *J*. *excelsa* M. Bieb [44, 52], and also its efficacy in the treatment of CL in an animal model [53]. Therefore, the current randomized controlled clinical trial has evaluated the efficacy of the leaf extract of this native plant on human CL for the first time.

Nowadays, there is an increase in patient interest for complementary and alternative medicine (CAM). Therefore, there is an essential need for evidence-based assessment of different CAM modalities, including herbal medicines [54–56]. According to medical and pharmaceutical manuscripts in traditional Persian medicine, CL appears as a dry or wet wound caused by insect (sand-fly) bites [5, 57]. This disease was described as *Rish-e-Balkhi* (Balkh Wound) or *Balkhi'ye* in traditional Persian medicine. The medieval descriptions and signs and symptoms of this wound is very close to what is called cutaneous leishmaniasis in current medicine [58, 59]. *J*. *excelsa* which is known as *Abhol* or *Urs* in *Makhzan al-adviyah* (the Storehouse of Medicament authored by Aghili Shirazi in 18^th^ century A.D.), was administered for the treatment of severe infected wounds called *Qūrūh-e-khabisa* (non-healing wounds) [60]. Abu Mansour Heravi, the author of "*al Abnieh an-Haghayegh al-Advieh*" (10^th^ century A.D.) [61, 62], prescribed *Abhol* for the treatment of infected wounds and also fresh wounds as well as *Rish-e-balkhi* [63].

Effectiveness of *J*. *excelsa* in the healing process of CL is related to the presence of various classes of metabolites in the extracted sample. Some of these are mentioned here: *J*. *excelsa* is a plant rich in phenols, terpenoids and flavonoid components [64]. Several studies have represented the remarkable antileishmanial activities of phenols, flavonoids or terpenoids in various extracts [65, 66]. The underlying mechanism of phenolic compounds and flavonoids is related to the release of lacyate dehydrogenase by macrophages which results in the antileishmanial activity of *J*. *excelsa* [67, 68]. The antioxidant and anti-inflammatory activity of JE can lead to acceleration in healing of chronic ulcers like CL. Antioxidants have an important role in suppression of the oxidative processes in the early phase of wound healing. However, antioxidative mechanisms are based on gradual detoxification of free radicals and oxidative agents and on gradual return of cells to the state of redox homeostasis [69, 70].

Tendency to chronicity of CL, is the most important reason for the treatment of this disease [12]. There are several recommended remedies in the treatment of CL including: cryotherapy and thermotherapy as physical methods [26, 71], topical and systemic medications and herbal remedies and natural products [72, 73].

Antimonials, are the most common reagents used to treat CL since 50 years ago in both intra-lesional and systemic form. Many patients refuse to use these medicaments due to possible pain and or other adverse effects. Also some physicians dislike to prescribe antimonials due to some important systemic and local side effects or absolute or relative contraindications [74, 75]. On the other hand, there is some evidence revealing the presence of some resistant strains of CL to meglumine antimoniate in recent years [76, 77]. Therefore, finding alternative remedies with less complications are necessary to manage this disease.

Cryotherapy, is the most local standard therapeutic method for the treatment of CL with variable efficacy [29]. Using this method is accompanied with a painful sensation and has several mild to serious complications. On the other hand, the wound may become susceptible to infection due to the induced necrosis and secondary ulceration of the tissue [78, 79]. Several studies have demonstrated that combination therapy in the treatment of CL can improve the treatment duration [80] and accordingly, the combination of cryotherapy and intra-lesional meglumine antimoniate, can increase the efficacy up to 89% [20].

We conducted the first triple blind randomized clinical trial to compare the efficacy of combination therapy of a topical cream containing *J*. *excelsa* leaf hydroalcoholic extract in association with cryotherapy versus cryotherapy and placebo in CL patients. Overall, 82% of patients in the treatment group (group A) had complete cure, and 9% of them showed partial cure. In comparison with patients in the placebo group (group B), the result of our study confirmed that topical 5% cream of JE applied three times daily for around three months was more effective compared to cryotherapy plus placebo. These results are in-line with the wound healing effect of *J*. *excelsa* in traditional Persian medicine sources, as *Makhzan al adviyah* [60, 81]. Outcomes of our study is close to the effectiveness of combination therapy of meglumine antimoniate and cryotherapy in Salmanpour’s study [20].

Several investigations have demonstrated the therapeutic effects of *J*. *excelsa* in various pharmacological modalities. One study has revealed the anti-inflammatory effect of the plant’s subtypes [82]. Other studies demonstrated anti-parasitic, anti-fungal and anti-microbial effect of *J*. *excelsa* essential oil or hydroalcoholic extract, which is in relation with the presence of potent fractions or metabolites in the essential oil or extracts of this plant [81, 83]. Anti-tumor and cytotoxic activity of *J*. *excelsa* leaves and fruit essential oil have also been shown in other studies [84]. *Persian Juniper* has been shown to have an effect on cell-cycle phases of MCF-7 breast cancer cell line [85]. Satisfactory antileishmanial activity of *Juniperus* species have also been demonstrated in a few in-vitro and in-vivo studies [44, 53, 86]. In addition, antioxidant and radical scavenging activities of high content of terpenoids in essential oil and considerable phenolic content in extracts of *J*. *excelsa* have been indicated useful for wound healing [87–89]. Mirzavand et al reported that the extract of the leaf of *J*. *excelsa* has more inhibitory effects on *Leishmania* amastigote in comparison with meglumine antimoniate in an animal model. In that study, the reduction of diameter of the lesions became statistically significant in the sixth week of treatment in the treatment group with *J*. *excelsa* as compared to that of the control group. However, there were no statically significant differences in reduction size of mice lesions in the *J*. *excelsa* group in comparison with the intra-lesional meglumine antimoniate group [53].

There are also several plants which have been examined for antileishmanial activity. Zerehsaz et al, showed the therapeutic effect of Z-HE (a traditional medicine) in healing of CL[90]. Another study demonstrated the anti-leishmanial effect of *Peganum harmala* L [91]. Chan-Bacab *et al* showed that *Annona senegalensis* Pers possessed several components with antileishmanial activity against related promastigotes [92]. Additional in-vitro and in-vivo studies revealed the leishmanicidal activity and cytotoxicity of extracts of *Ricinus communis* L. and *Azadirachta indica* A.Juss., especially in combination therapy [93].

In our study, five patients experienced local irritation after five to nine weeks of the JE administration in group A. However, this difference was not statistically significant as compared to that of group B. Overall, no acute reaction was observed. To our knowledge, this is the first report of any side effects related to the use JE in the medical literature.

The result of GEE model in our study demonstrated that combination of cryotherapy with a cream containing *J*. *excelsa* hydroalcoholic extract can decrease the duration of CL treatment and also result in a decrease in the number of cryotherapy sessions. Therefore, it seems that a cream containing JE in combination with cryotherapy in the treatment of CL can be a good alternative to meglumine antimoniate.

In our study, except for one authentication of leishmania species as *L*. *infantum*, all cases with positive PCR, were identified as *L*. *major*. Predominancy of the *L*. *major* subtype in Iran has been reported in previous studies [10, 94, 95]. The *L*. *infantum* case was in group B, which received cryotherapy plus placebo. Therefore, we did not evaluate the effect of the JE, according to the species of CL, in this study. It is interesting to note that the patient with *L*.*infantum* was labeled as failure to treatment at the 12th-week of follow-up.

The PCR method, did not identify the strain of *Leishmania* amastigote in a few of the patients who had positive smears for *Leishmania* amastigote. This finding may be due to technical errors in sampling of the tissue and/or processing of the PCR. On the other hand, it is worth mentioning that the sensitivity of PCR for detection of leishmaniasis is not perfect [96]. Four patients out of 33 in group A and one out of 29 patients in group B (a total of 5 patients with PCR-undetected strains of *Leishmania)*, had complete cure in this study. The other 4 patients (one patient in group A and 3 patients in group B), with PCR-undetected strains of *Leishmania*, failed treatment.

### Limitations of the study

Firstly, our project is a combination study. Both groups received cryotherapy as baseline therapy. As cryotherapy was done 1–2 millimeters around the lesions, the size of the lesions were affected with this procedure, and it can be a confounding factor in both groups. Secondly, dose assessment was not performed in our study. In other words, we did not evaluate different concentrations of the JE in the cream base. Also, we excluded the patients who had CL lesions on the face due to ethical considerations. Therefore, we could not evaluate the effect of this herbal extract on facial ulcers and subsequently, hypersensitivity or side effects on the facial skin. Moreover, children under 18 years of age were excluded from this study due to ethical issues. In this regard, we could not evaluate this product in children. We expect that the response in children will be acceptable due to a thinner skin and easier penetration, in comparison with adults. Furthermore, we could not evaluate the serum level of active constituents of JE in order to estimate the dermal absorption of this extract.

## Conclusion

The result of this study demonstrated a good efficacy of JE in a 5% cream base, for the treatment of CL. Since JE, is a cheap, easy available and safe product, it can be considered as an alternative adjuvant treatment modality in CL. Overall, we suggest further studies in order to focus on mono-therapy with this preparation (prescribing only the cream containing JE without any concomitant modalities), in the treatment of CL.

## Supporting information

S1 Consort Checklist (DOCX)

S1 Fig CONSORT chart of the clinical trial of therapeutic effect of *Juniperus excelsa* M. Bieb extract cream on cutaneous leishmaniasis. (DOCX)

S2 Fig Electrophoresis of PCR products of DNA extracted from positive smears. The 15 lanes are shown in this figure and consist of: ladder lanes (1 and 15); weakly positive (lane 2); positive control of *L*. *infantum* (lane 3); positive control of *L*. *major* (lane 14); Patients samples (lanes 4–13).
(DOCX)

S3 Fig Changes in the size of CL lesions in both groups during three months. The numbers on the left side of the chart indicate the changes of length of the ulcers (mm) and the numbers on right side of the chart indicate the area changes (mm^2^) in the duration of treatment (12 weeks).
(DOCX)

S4 Fig CL patient who was cured in group A. (A)Before treatment, (B)after one week, (C) after two weeks, (D)after three weeks, (E) after five weeks.
(DOCX)

S1 Table Demographic characteristics of CL patients in both groups (Group A and Group B). (DOCX)

S2 Table Outcome of the treatment in both groups (Group A and Group B). (DOCX)

S3 Table Volatile constituents of both the plant methanol extract and prepared JE cream. The significance of bold is to present the most abundant constituents. Compounds have been identified by combination of both mass spectra and retention indices. RI represents the retention indices which were calculated against C8-C24 n-alkanes in the mentioned column. Compounds have been sorted with respect to retention indices on HP-5 MS capillary column.
(PDF)
