# Supplementary material for: Efficacy of cryotherapy plus topical Juniperus excelsa M. Bieb cream versus cryotherapy plus placebo in the treatment of Old World cutaneous leishmaniasis: A triple-blind randomized controlled clinical trial
Source: PLoS Negl Trop Dis. 2017 Oct 5;11(10):e0005957. doi: 10.1371/journal.pntd.0005957 (PMC5655399; doi:10.1371/journal.pntd.0005957)

**S1 Fig -** CONSORT chart of the clinical trial of therapeutic effect of *Juniperus excelsa* M. Bieb extract cream on cutaneous leishmaniasis.


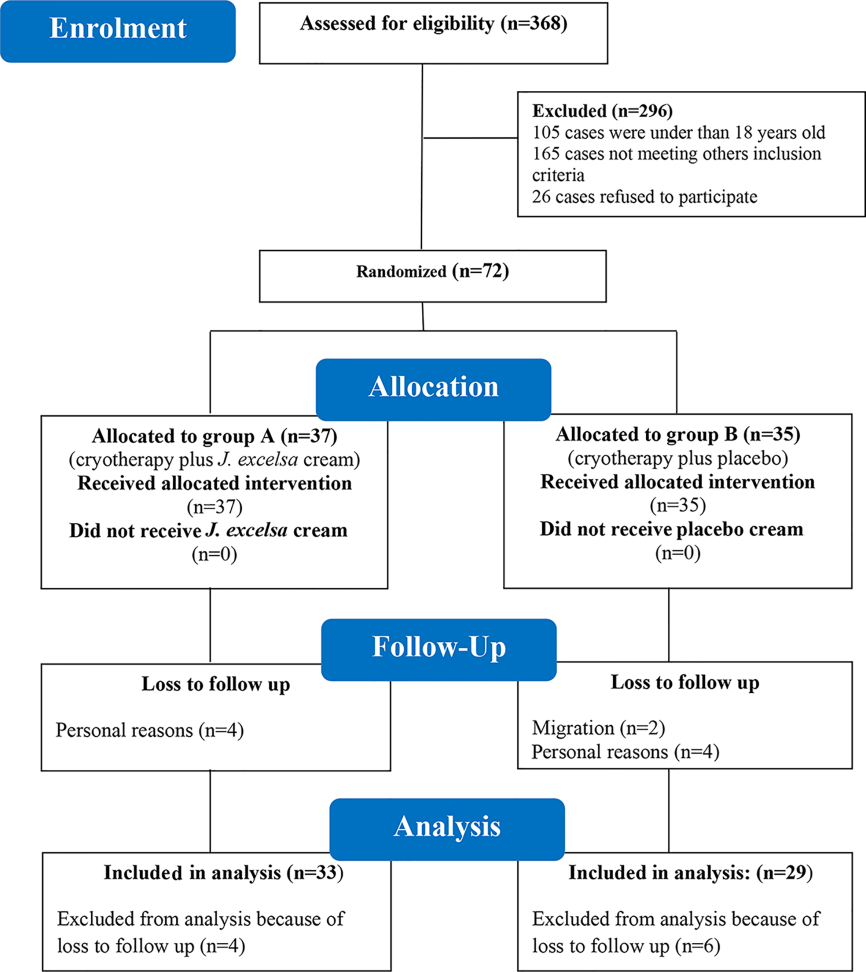

Supplement: S1 Fig — (DOCX) [file pntd.0005957.s002.docx]
