# Supplementary material for: Efficacy of cryotherapy plus topical Juniperus excelsa M. Bieb cream versus cryotherapy plus placebo in the treatment of Old World cutaneous leishmaniasis: A triple-blind randomized controlled clinical trial
Source: PLoS Negl Trop Dis. 2017 Oct 5;11(10):e0005957. doi: 10.1371/journal.pntd.0005957 (PMC5655399; doi:10.1371/journal.pntd.0005957)

**S2** **Fig - Electrophoresis of PCR products of DNA extracted from positive smears.** The 15 lanes are shown in this figure and consist of: ladder lanes (1 and 15); weakly positive (lane 2); positive control of *L. infantum* (lane 3); positive control of *L. major* (lane 14); Patients samples (lanes 4-13).


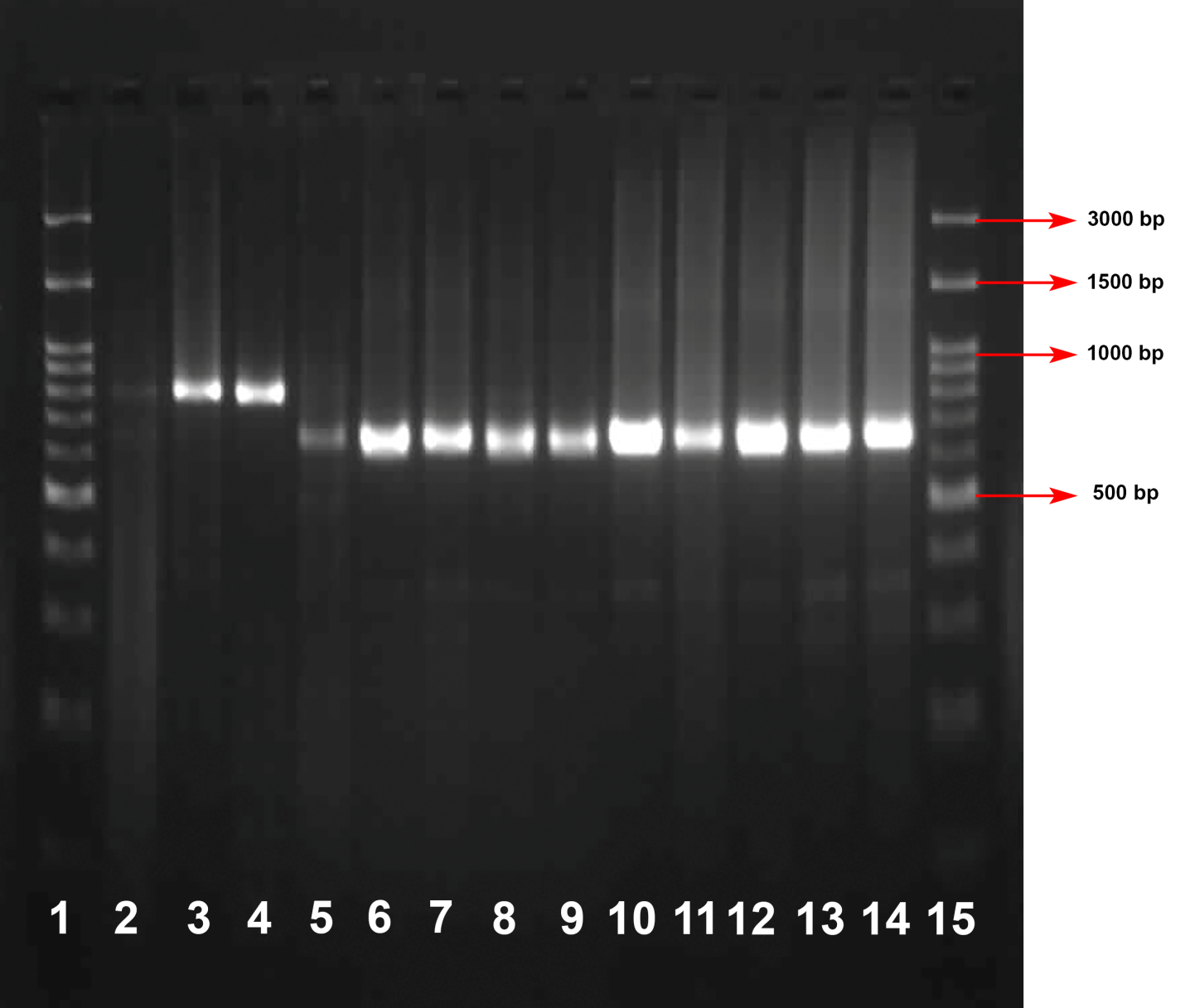

Supplement: S2 Fig — The 15 lanes are shown in this figure and consist of: ladder lanes (1 and 15); weakly positive (lane 2); positive control of L. infantum (lane 3); positive control of L. major (lane 14); Patients samples (lanes 4–13). (DOCX) [file pntd.0005957.s003.docx]
