# Supplementary material for: Efficacy of cryotherapy plus topical Juniperus excelsa M. Bieb cream versus cryotherapy plus placebo in the treatment of Old World cutaneous leishmaniasis: A triple-blind randomized controlled clinical trial
Source: PLoS Negl Trop Dis. 2017 Oct 5;11(10):e0005957. doi: 10.1371/journal.pntd.0005957 (PMC5655399; doi:10.1371/journal.pntd.0005957)

**S3 Fig - Changes in the size of CL lesions in both groups during three months**. The numbers on the left side of the chart indicate the changes of length of the ulcers (mm) and the numbers on right side of the chart indicate the area changes (mm^2^) in the duration of treatment (12 weeks)


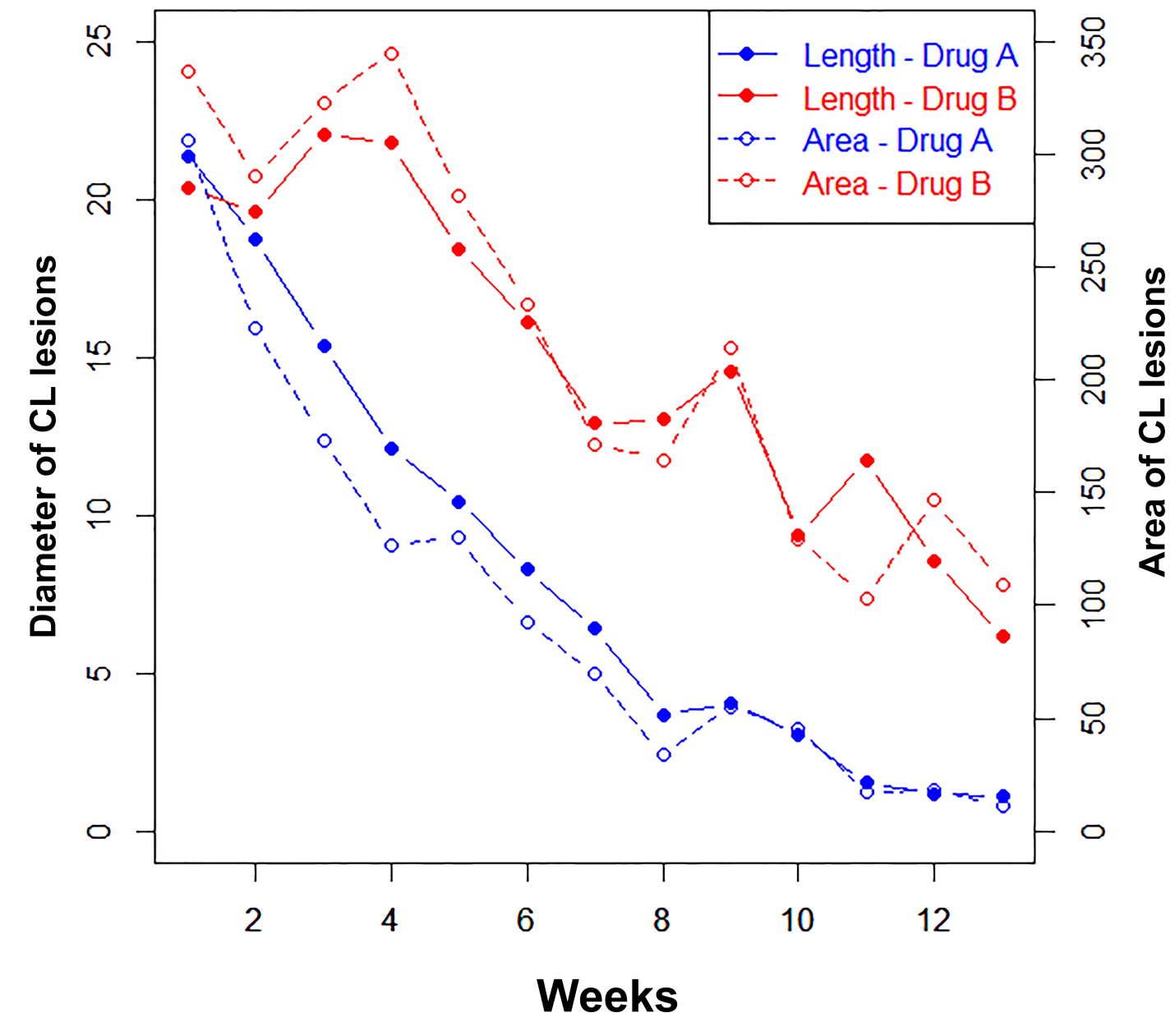

Supplement: S3 Fig — The numbers on the left side of the chart indicate the changes of length of the ulcers (mm) and the numbers on right side of the chart indicate the area changes (mm2) in the duration of treatment (12 weeks). (DOCX) [file pntd.0005957.s004.docx]
