# Supplementary material for: Efficacy of cryotherapy plus topical Juniperus excelsa M. Bieb cream versus cryotherapy plus placebo in the treatment of Old World cutaneous leishmaniasis: A triple-blind randomized controlled clinical trial
Source: PLoS Negl Trop Dis. 2017 Oct 5;11(10):e0005957. doi: 10.1371/journal.pntd.0005957 (PMC5655399; doi:10.1371/journal.pntd.0005957)

**S4 Fig -**  **CL patient who was cured in group A.** (A)Before treatment, (B)after one week, (C) after two weeks, (D)after three weeks, (E) after five weeks.


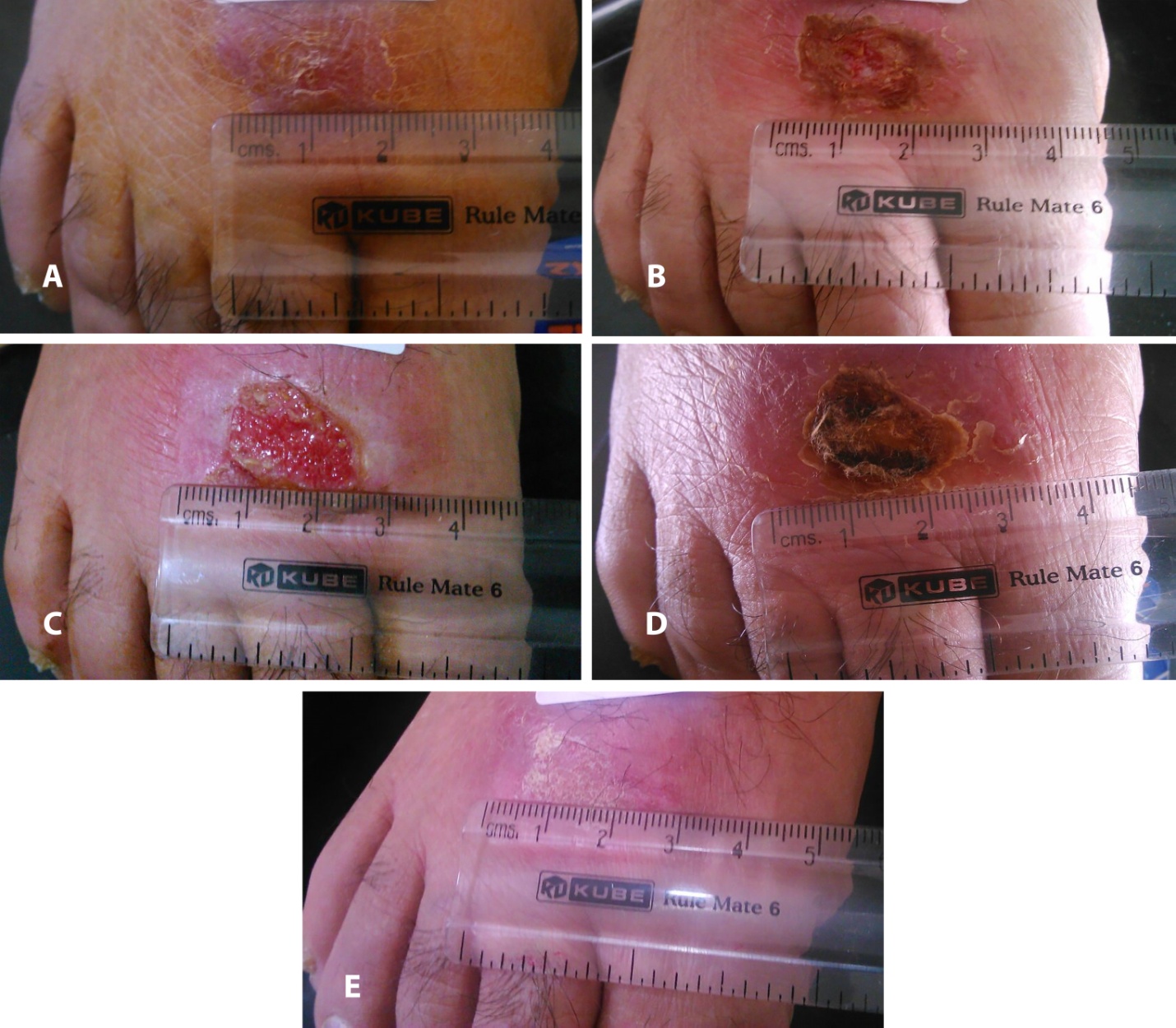

Supplement: S4 Fig — (A)Before treatment, (B)after one week, (C) after two weeks, (D)after three weeks, (E) after five weeks. (DOCX) [file pntd.0005957.s005.docx]
