# Supplementary material for: Efficacy of cryotherapy plus topical Juniperus excelsa M. Bieb cream versus cryotherapy plus placebo in the treatment of Old World cutaneous leishmaniasis: A triple-blind randomized controlled clinical trial
Source: PLoS Negl Trop Dis. 2017 Oct 5;11(10):e0005957. doi: 10.1371/journal.pntd.0005957 (PMC5655399; doi:10.1371/journal.pntd.0005957)
Supplement: S1 Table — (DOCX) [file pntd.0005957.s006.docx]

**Table 1-** Demographic characteristics of CL patients in both groups (Group A and Group B)

| **Variables** | **Cryotherapy plus JE** | **Cryotherapy plus placebo** | **P-value** |
| --- | --- | --- | --- |
|  | **( Group A)** | **( Group B)** |  |
| **Sex** |  |  | 0.49 |
| Male | 21 (67%) | 16 (55%) |  |
| Female | 12 (36%) | 13 (45%) |  |
| **Age** (year , mean± SD) | 38.91±13.49 | 42.10±14.54 | 0.437 |
| **Marriage status** |  |  | 0.24 |
| Single | 9 (27%) | 12 (41%) |  |
| Married | 24 (73%) | 17 (57%) |  |
| **Educational status** |  |  | 0.53 |
| Under-Diploma | 21 (64%) | 17 (59%) |  |
| Diploma | 5 (15%) | 7 (24%) |  |
| Associate Degree | 3 (9%) | 2 (7%) |  |
| Bachelor Degree | 4 (12%) | 3 (10%) |  |
| **Location of the lesions** |  |  | 0.61 |
| Upper extremity | 19 (58%) | 16 (55%) |  |
| Lower extremity | 11 (33%) | 8 (28%) |  |
| Both upper and lower extremities | 3 (9%) | 5 (17) |  |
| **Number of the lesions** |  |  | 0.65 |
| 1 | 18 (55%) | 17 (57%) |  |
| 2 | 8 (24%) | 5 (17%) |  |
| 3 | 5 (15%) | 3 (10%) |  |
| 4 | 2 (6%) | 4 (14%) |  |
| **PCR characterization of microscopic positive samples** |  |  | 0.85 |
| *Leishmania major* | 28 (85%) | 24 (83%) |  |
| *Leshmania infantum* | 0 (0%) | 1 (3%) |  |
| *Leshmania tropica* | 0 (0%) | 0 (0%) |  |
| Negative result of PCR | 5 (15%) | 4 (14%) |  |
| **Duration between time of lesion occurrence and the time of first visit for treatment** (month, mean± SD) | 1.56±0.74 | 1.44±0.83 | 0.34 |
| **Vertical diameter of lesion on first visit**(mm, mean± SD) | 21.38± 7.87 | 20.41±7.95 | 0.65 |
| **Horizontal diameter of lesion on first visit** (mm, mean± SD) | 16.66± 5.63 | 17.52± 7.05 | 0.6 |
| **Area of the lesion**  (mm^2^, mean± SD) | 306.26±226.60 | 336.95±303.32 | 0.94 |

**JE*:*** *Juniperus excelsa* M. Bieb *extract*, **mm:** millimeter; **mm^2^**: square millimeter; SD: standard deviation; PCR: Polymerase chain reaction
