# Supplementary material for: Efficacy of cryotherapy plus topical Juniperus excelsa M. Bieb cream versus cryotherapy plus placebo in the treatment of Old World cutaneous leishmaniasis: A triple-blind randomized controlled clinical trial
Source: PLoS Negl Trop Dis. 2017 Oct 5;11(10):e0005957. doi: 10.1371/journal.pntd.0005957 (PMC5655399; doi:10.1371/journal.pntd.0005957)
Supplement: S2 Table — (DOCX) [file pntd.0005957.s007.docx]

**Table 2-** Outcome of the treatment in both groups (Group A and Group B)

| **Variable** | **Cryotherapy plus JE** | **Cryotherapy plus placebo** | **P-value** |
| --- | --- | --- | --- |
|  | **( Group A)** | **( Group B)** |  |
| **Result of treatment**  N (%) |  |  | <0.001^*^ |
| Complete cure | 27 (82%) | 10 (34%) |  |
| Partial cure | 3 (9%) | 4 (14%) |  |
| Failure to treatment | 3 (9%) | 15 (52%) |  |
| **Duration to cure** | 6.48±2.96 | 8.72±3.34 | 0.04* |
| (mean± SD of weeks) |  |  |  |
| **Drug reaction**  N (%) |  |  | 0.055 |
| No | 28 (85%) | 29 (100%) |  |
| Yes | 5 (15%) | 0 (0%) |  |
| **Number of cryotherapy sessions in patients with complete cure** (mean± SD) | 3.85±2.03 | 6.54±3.35 | 0.026* |

*Significant at 5%, **JE*:*** *Juniperus excelsa* M. Bieb *extract*, **SD:** standard deviation
