# Supplementary material for: Efficacy of cryotherapy plus topical Juniperus excelsa M. Bieb cream versus cryotherapy plus placebo in the treatment of Old World cutaneous leishmaniasis: A triple-blind randomized controlled clinical trial
Source: PLoS Negl Trop Dis. 2017 Oct 5;11(10):e0005957. doi: 10.1371/journal.pntd.0005957 (PMC5655399; doi:10.1371/journal.pntd.0005957)
Supplement: S3 Table — The significance of bold is to present the most abundant constituents. Compounds have been identified by combination of both mass spectra and retention indices. RI represents the retention indices which were calculated against C8-C24 n-alkanes in the mentioned column. Compounds have been sorted with respect to retention indices on HP-5 MS capillary column. (PDF) [file pntd.0005957.s008.pdf]

Table 3- Volatile constituents of both the plant methanol extract and prepared JE cream[1]

No.	Component	Area % (Extract)	Area % (Cream)	KI^C	KI^R
1	α-Pinene	-	0.24	935	939
2	Limonene	-	0.21	1030	1029
3	Linalool	1.02	0.34	1100	1096
4	trans-Pinocarveol	-	0.27	1142	1139
5	Borneol	0.36	0.38	1168	1168
6	α- Terpineol	0.31	-	1193	1188
7	n-Dodecane	-	0.45	1200	1200
8	n-Decanol	**-**	0.19	1272	1269
9	Thymol	0.71	-	1291	1290
10	n-Tridecane	-	0.53	1300	1300
11	Carvacrol	1.55	-	1301	1290
12	α-Cubebene	0.5	-	1353	1348
13	Hexyl hexanoate	-	0.3	1387	1383
14	n-Tetradecane	-	0.83	1400	1400
15	β-Funebrene	-	1.37	1419	1414
16	trans-Caryophyllene	-	0.32	1425	1419
17	α-Humulene	-	0.37	1459	1454
18	n-Dodecanol		2.74	1477	1472
19	γ-Muurolene	0.53	0.22	1478	1479
20	α-Amorphene	0.71	-	1481	1484
21	Germacrene D	0.53	-	1497	1485
22	n-pentadecane	-	1.63	1503	1500
23	α-Muurolene	1.72		1505	1500
24	ɣ-Cadinene	1.51		1519	1513
25	Butylated hydroxytoluene	**-**	**34.74**	1524	1515
26	Methyl dodecanoate	1.45	-	1525	1525
27	Δ-Cadinene	4.42	0.65	1528	1523
28	Elemol	**13.36**	0.7	1555	1549
29	Germacrene B	-	0.45	1564	1561
30	Dodecanoic acid	1.78	-	1570	1566
31	Spathulenol	0.6	0.73	1582	1578
32	Caryophyllene oxide	0.83	-	1588	1583
33	Ethyl dodecanoate	0.42	-	1593	1595
34	n-Hexadecane	-	1.37	1600	1600
35	β-Oplopenone	1.23		1613	1607
36	Cedrol	**-**	**13.66**	1613	1619
37	α -Cadinol	**7.9**	1.17	1636	1640
38	β -Eudesmol	**7.51**	0.79	1647	1650
39	α-Eudesmol	**30.02**	0.46	1660	1653
40	n-Heptadecane	1.16	0.87	1698	1700
41	Methyl tetradecanoate	0.91		1723	1723
42	n-Octadecane	-	0.64	1797	1800
43	n-Hexadecanol	**-**	**19.99**	1886	1875

44	n-Nonadecan	-	0.24	1898	1900
45	Methyl hexadecanoate	0.84	-	1923	1921
46	Hexadecanoic acid	1.26	-	1958	1960
47	n-Eicosane	0.69	0.19	1996	2000
48	n-octadecanol	**-**	**8.72**	2085	2077
49	Methyl linoleate	0.39	-	2091	2085
	**Total identification**	**84.22**	**95.76**	-	-
	**Monoterpenes**	**3.95**	**1.1**	-	-
	**Sesquiterpenes**	**75.02**	**21.29**	-	-
	**Phenols**	**-**	**34.74**		
	**Hydrocarbons**	**5.25**	**38.63**	-	-

[1]The significance of bold is to present the most abundant constituents. Compounds have been identified by combination of both mass spectra and retention indices. RI represents the retention indices which were calculated against C8-C24 n-alkanes in the mentioned column. Compounds have been sorted with respect to retention indices on HP-5 MS capillary column.
